# Respiratory modulation of neurophysiology and symptoms in athletes with sports-related concussion: a randomized crossover trial

**DOI:** 10.3389/fnins.2026.1825070

**Published:** 2026-06-17

**Authors:** Wendi Wang, Hongyu Zhu, Xiaolin Gao, Tao Yu, Xin Yan, Gonghao Wang, Zhiqiang Zhang

**Affiliations:** 1 School of Exercise and Health, Shanghai University of Sport, Shanghai, China; 2 Sports Rehabilitation Research Center, China Institute of Sport Science, Beijing, China; 3 China Swimming Association, Beijing, China; 4 China Agricultural University, Beijing, China

**Keywords:** autonomic nervous system, electroencephalography, pelvic floor resonance breathing, resonance breathing, skin sympathetic nerve activity, sports-related concussion

## Abstract

**Objectives:**

This study examined the effects of two breathing interventions on athletes with persistent post-concussive symptoms. Resonance breathing (RB) is a slow breathing technique at an individual’s resonance frequency. Pelvic floor resonance breathing (PRB) combines diaphragmatic breathing with pelvic floor muscle activation.

**Methods:**

A randomized crossover trial was conducted using a 3–1–3 week design. Two intervention phases were separated by a 1-week washout period. Twenty-six male rugby athletes with persistent post-concussive symptoms were included. Participants were assigned to different intervention sequences. Outcomes included the Rivermead Post-Concussion Symptoms Questionnaire, electroencephalography (EEG), heart rate variability (HRV), and skin sympathetic nerve activity (SKNA). Measurements were taken at baseline and after each intervention.

**Results:**

Total symptom scores decreased after both interventions (*p* < 0.01). Somatic symptom scores were lower after PRB (*p* < 0.05), whereas emotional symptom scores were lower after RB (*p* < 0.05). EEG results showed different pre-post patterns between interventions. PRB was associated with broader pre-post changes in *β* and *θ* bands, whereas RB showed more localized pre-post changes in β, θ, and *δ* bands. Heart rate was lower and HRV total power was higher after both interventions (*p* < 0.05). The LF/HF ratio and SKNA burst area were higher after RB (*p* < 0.05), whereas SKNA burst threshold and frequency were higher after PRB (*p* < 0.05). Correlation analysis suggested an association between prefrontal activity and SKNA changes in PRB.

**Conclusion:**

Both RB and PRB were associated with symptom improvement and changes in neuro-autonomic function. The two interventions showed different patterns. These findings support the use of breathing interventions as non-pharmacological approaches in concussion rehabilitation.

## 1 Introduction

Sports-related concussion (SRC) is a prevalent form of mild traumatic brain injury in contact-sport athletes (McCrory et al., 2017). Although symptoms vary across individuals, some athletes experience persistent post-concussive symptoms (PCS) beyond the expected recovery period (Krzyzaniak and Fatehi Hassanabad, 2022). PCS involves interacting neurometabolic, neuroinflammatory, cerebrovascular, and autonomic disturbances (Giza and Hovda, 2001). Autonomic dysfunction following SRC has been widely reported, but its manifestations differ across studies, with evidence of sympathetic predominance, parasympathetic withdrawal, or mixed patterns (Abaji et al., 2016; Hilz et al., 2011; Senthinathan et al., 2017). Therefore, frequency-domain HRV indices, including LF, HF, and LF/HF ratio, were selected to characterize autonomic modulation because they are widely used non-invasive markers of cardiovascular autonomic regulation in both general physiological research and sport-related concussion studies, although the LF/HF ratio should be interpreted cautiously (Task Force of the European Society of Cardiology and the North American Society of Pacing and Electrophysiology, 1996; Bishop et al., 2018; Shaffer and Ginsberg, 2017). Persistent symptoms can impair quality of life and athletic performance, while current management relies mainly on relative rest, graded return-to-activity strategies, and symptom-targeted pharmacological support when needed (Kushner, 1998; Leddy et al., 2016; Pertab et al., 2018). Thus, additional non-invasive approaches are needed to support recovery.

Respiration, a fundamental vital function, is a whole-body process accomplished through the highly coordinated, rhythmic alternation of muscle contraction and relaxation, primarily involving the thoracic and abdominal cavities (Cook et al., 2021). Resonance breathing (RB) is a slow-paced breathing exercise performed at an individual’s resonance frequency and has been used in neurorehabilitation contexts through autonomic modulation (Conder et al., 2020). Pelvic floor resonance breathing (PRB) combines slow breathing with coordinated abdominal and pelvic floor muscle activation, which may increase intra-abdominal pressure and somatic-autonomic engagement (Meraklı, 2025; Tatschl et al., 2022). However, PRB has not yet been systematically examined in SRC rehabilitation.

To better evaluate the potential of PRB as a novel neuromodulatory intervention for sports-related concussion (SRC), further consideration of the neural pathways linking respiratory mechanics to cortical activity is warranted. Importantly, slow breathing has been shown to modulate autonomic nervous system activity, particularly by enhancing parasympathetic (vagal) tone through cardiorespiratory coupling (Lehrer and Gevirtz, 2014; Russo et al., 2017; Zaccaro et al., 2018). This autonomic modulation can influence central autonomic networks, including regions such as the prefrontal cortex, thalamus, and brainstem, which play key roles in the generation and regulation of cortical oscillations (Benarroch, 1993; Beissner et al., 2013; Buzsáki and Draguhn, 2004). In addition, breathing-based interventions have been associated with changes in EEG activity, including increased frontal alpha power and reduced theta activity, which are often linked to improved emotional and cognitive regulation (Bing-Canar et al., 2016; Ma et al., 2017; Zaccaro et al., 2018). Since SRC often affects these specific neurological networks, it is plausible that respiratory biofeedback may influence EEG outcomes indirectly through autonomic and central neural mechanisms in athletic populations (Pertab et al., 2018; Romeu-Mejia et al., 2019). Furthermore, rigorous clinical validation of its efficacy is lacking (Leddy et al., 2021).

Therefore, this study investigated PRB as a targeted breathing intervention for athletes with SRC and examined its associations with symptoms, autonomic function, SKNA, and EEG outcomes. This work may provide preliminary evidence for respiratory biofeedback as an adjunctive, non-invasive rehabilitation approach.

## 2 Methods

### 2.1 Participants

Male athletes from a Beijing rugby team were screened for eligibility based on the following criteria: (1) aged 18–35 years; (2) possession of a national athlete certification of Grade II or above; (3) a history of significant impact to the head, neck, or face during sports, immediately followed by signs of impaired neurological function (e.g., headache, confusion, dizziness, amnesia, or transient loss of consciousness); (4) presence of at least three persistent post-concussive symptoms (lasting ≥14 days); and (5) clinical stability at recruitment, no use of psychotropic medications, no prior extensive breath training (>1 year), and willingness to provide informed consent and complete the entire trial protocol.

Of the twenty-seven athletes who met the criteria and provided informed consent, one withdrew for personal reasons, yielding a final sample of 26 for analysis. The participants had the following characteristics (mean ± SD): age, 20.9 ± 3.7 years; height, 182.9 ± 4.64 cm; weight, 89.5 ± 11.35 kg; and BMI, 26.7 ± 2.89 kg/m^2^. The cohort comprised 14 Master Athletes, 7 Grade I athletes, and 5 Grade II athletes. The mean time from injury to recruitment was 4.3 ± 3.2 weeks. Injury mechanisms were categorized as: direct impact during competition (*n* = 18, 69.2%), direct impact during training (*n* = 6, 23.1%), and indirect impact (*n* = 2, 7.7%).

### 2.2 Experimental protocol

**Figure 1:**
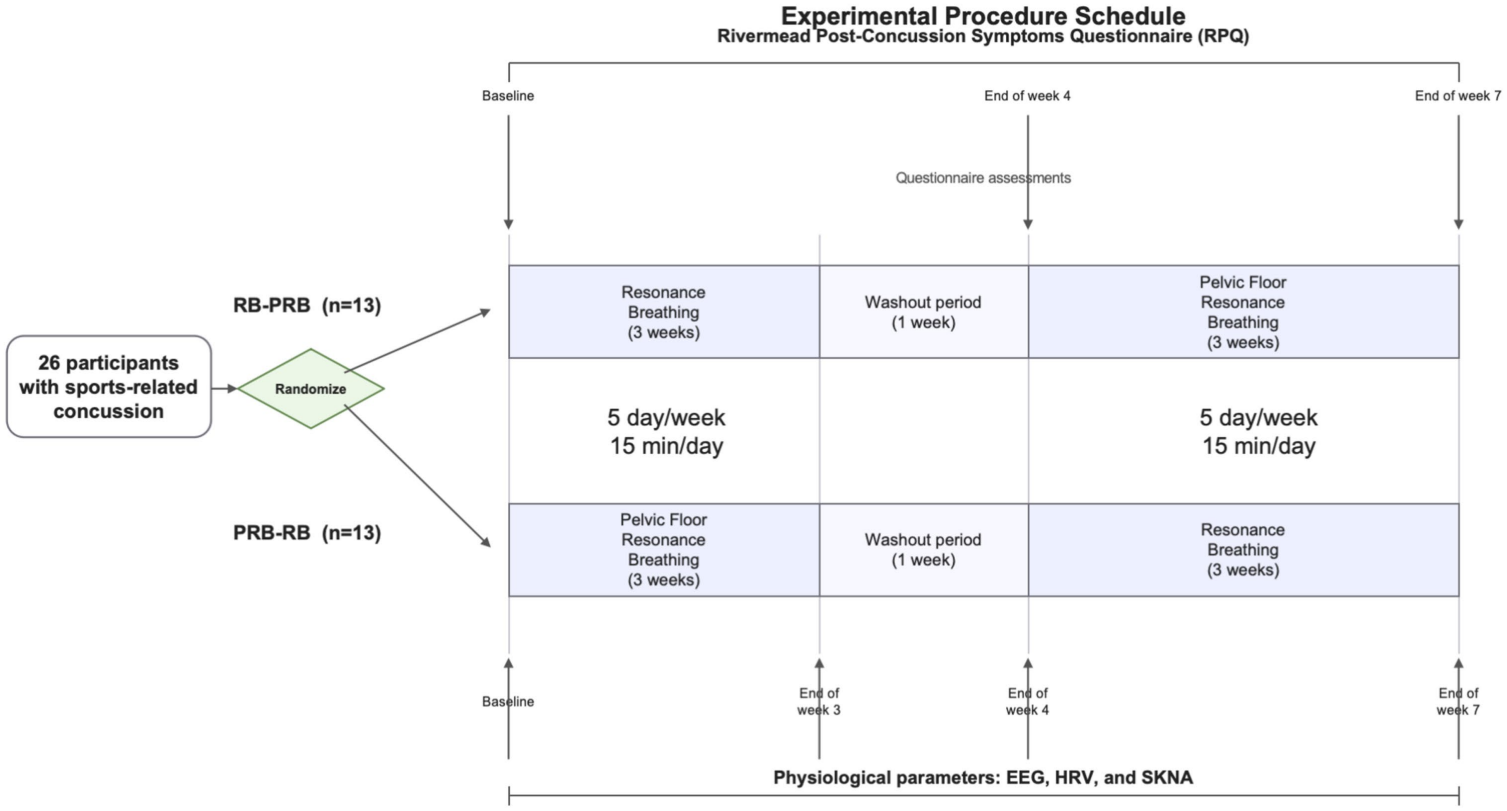
Experimental procedure schedule.

This study utilized a randomized crossover design. Twenty-six participants were randomly allocated to one of two intervention sequences (*n* = 13 per group). Group A commenced with 3 weeks of standard resonance breathing (RB) training, followed by a 1-week washout period, and subsequently received 3 weeks of pelvic floor resonance breathing (PRB) training. Group B received the same interventions but in the reverse order (i.e., PRB first, followed by RB). The total duration of the study was 7 weeks, and all participants were instructed to maintain their regular training and daily activities throughout this period. This study was approved by the Ethics Committee of the China Institute of Sport Science (Approval No.: 20240530). A schematic overview of the study timeline and data collection schedule is provided in Figure 1.

The intervention was administered using a blended online and offline model. All participants first received a one-time, in-person instruction session from a physical therapist to ensure proper technique. Following this initial phase, the training was transitioned to supervised online group sessions. These sessions were conducted daily at 21:00, each lasting 15 min, with a requirement of at least 5 sessions per week. A therapist was present in all online sessions to monitor performance and ensure adherence to the protocol.

Participants were asked to maintain their usual training routines throughout the study period and were confirmed to be outside their competitive season. They were instructed to abstain from caffeine and alcohol for at least 12 h prior to each testing session. Participants were also advised to obtain adequate sleep on the night before testing and to avoid large meals within 2 h of the session. All HRV and EEG measurements were conducted by the same operator in a quiet, temperature-controlled environment.

### 2.3 Training methods

#### 2.3.1 Standard resonance breathing (RB)

The RB protocol was adapted from the established method by Lehrer et al. (2013). Participants performed the breathing exercise in a supine position, adhering to a rhythm of 5 s for inhalation followed by 5 s for exhalation. To maintain simplicity in the practice, they were instructed to perform both inhalation and exhalation through the nose, avoiding pursed-lip breathing techniques.

#### 2.3.2 Pelvic floor resonance breathing (PRB)

The PRB technique was implemented based on the procedures outlined by Tatschl et al. (2022). Participants commenced PRB training only after demonstrating proficiency in the standard RB. The technique required a synchronized contraction of the PFM, which was initiated at the start of inhalation and sustained for the entire inspiratory phase. The contraction was then released precisely at the onset of exhalation, with the muscles remaining fully relaxed until the next inhalation cycle began.

**Figure 2:**
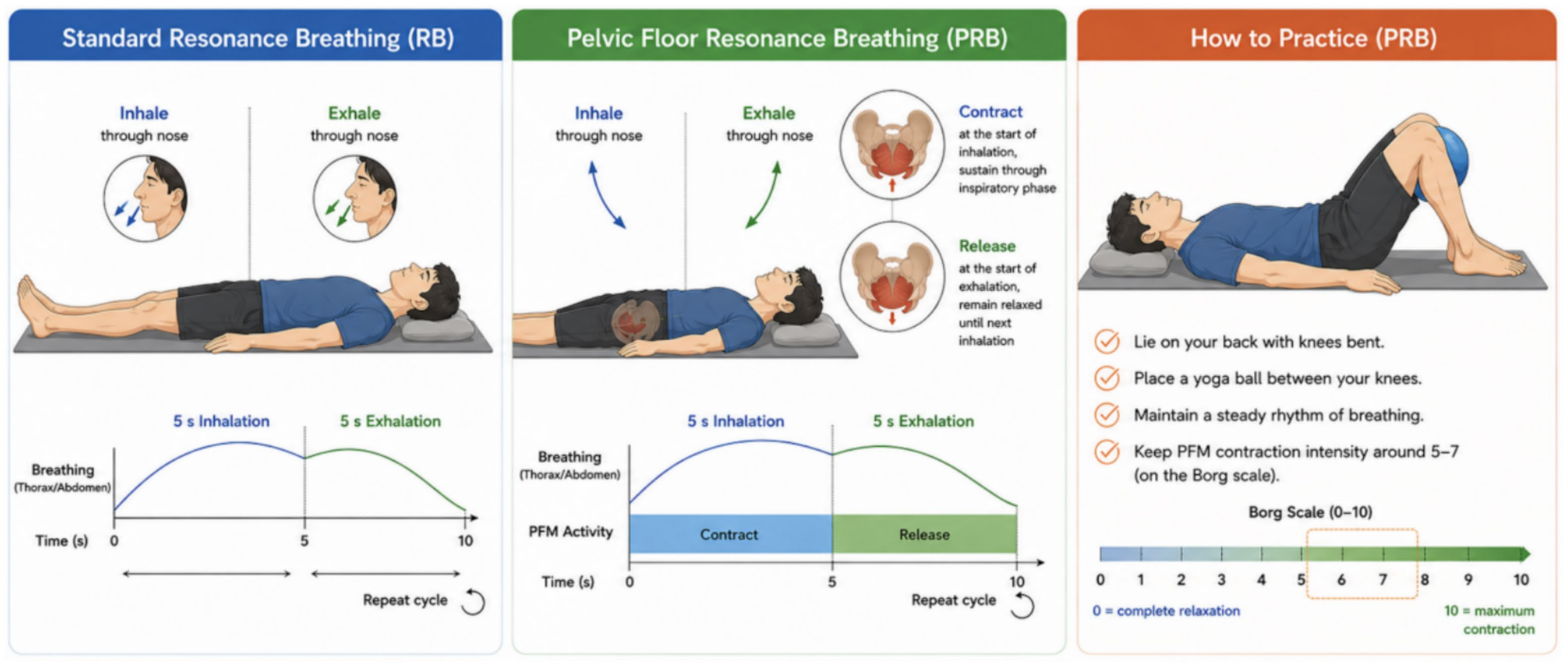
Training methods.

Contraction intensity was standardized using the Borg scale—a tool validated for assessing PFM activation (Stafford et al., 2016), where 0 represents complete relaxation and 10 indicates maximum voluntary contraction. Participants were guided to maintain a perceived intensity within the range of 5 to 7 (Stafford et al., 2015). For individuals who demonstrated difficulty with PFM isolation and control, an adjunct maneuver was employed. This involved adopting a supine position with lower limbs flexed and a training ball placed between the knees to augment proprioceptive feedback and facilitate correct muscle engagement. The training methods of RB and PRB are shown in the Figure 2.

### 2.4 Outcome measures

#### 2.4.1 Rivermead post-concussion symptoms questionnaire

Symptom severity was assessed using the Rivermead Post-Concussion Symptoms Questionnaire (RPQ). This 16-item scale covers somatic, cognitive, and emotional symptoms, each rated from 0 (not experienced) to 4 (severe). The total score ranges from 0 to 64, with a score ≥14 indicating clinically significant symptoms (Eyres et al., 2005).

#### 2.4.2 Electroencephalographic (EEG)

EEG signals were recorded using a QDBS1018 digital EEG system (Beijing Sun Technology Co., Ltd.) at a sampling rate of 125 Hz, which is sufficient for analyzing low-frequency EEG activity. Electrodes were positioned according to the international 10–20 system, a standardized method widely used in EEG research, with 19 channels recorded (Mecarelli, 2019). Raw EEG data were bandpass filtered using a finite impulse response (FIR) filter from 0.5 to 35 Hz to remove baseline drift and high-frequency noise, a range commonly applied in EEG preprocessing (Ishii et al., 2014). Artifacts, including electromyographic activity and movement-related noise, were visually inspected and manually removed to ensure data quality. Spectral analysis was performed using Fast Fourier Transform (FFT), and relative power was extracted as the primary frequency-domain measure. Frequency bands were defined based on established EEG conventions: delta (0.8–4 Hz), theta (4–7 Hz), alpha (8–13 Hz), and beta (13–20 Hz) (Ishii et al., 2014; Tang et al., 2019). Analysis focused on electrodes overlying the prefrontal cortex (Fp1, Fp2) and the dorsolateral prefrontal cortex (DLPFC; electrodes F3 and F4), regions commonly associated with cognitive control, emotional regulation, and autonomic integration (Benarroch, 1993; Thayer et al., 2009; Ochsner et al., 2012; Friedman and Robbins, 2022), and frequently examined in studies of brain responses to breathing and neuroregulatory interventions (Sherlin et al., 2010; Prinsloo et al., 2013).

#### 2.4.3 Autonomic function assessment

Autonomic nervous function was evaluated through heart rate variability (HRV) and skin sympathetic nerve activity (SKNA). A 5-min resting electrocardiogram was acquired in the supine position using a bio-signal acquisition system (ADInstruments Ltd., PLC01, New Zealand). Frequency-domain HRV metrics—including low-frequency (LF) power, high-frequency (HF) power, and the LF/HF ratio—were derived from the recorded data(Gullett et al., 2023). Simultaneously, SKNA signals were captured from the precordial area (Everett IV et al., 2017). Using the cyclic measurement feature in LabChart, SKNA parameters were computed from the 5-min resting neural recordings to quantify sympathetic activity intensity and burst characteristics. A sympathetic burst was defined as an SKNA signal peak exceeding 1.0–1.2 standard deviations above the mean value (Chen et al., 2023). The extracted SKNA metrics encompassed mean SKNA amplitude, burst threshold, frequency, amplitude, duration, and total burst area (Doytchinova et al., 2017).

### 2.5 Statistical analysis

Statistical analyses were performed using SPSS (version 26.0; IBM Corp., Armonk, NY, USA). Continuous variables were assessed for normality using the Shapiro–Wilk test. Data are presented as mean ± standard deviation (SD) for normally distributed variables, or median (interquartile range, IQR) for non-normal data. RPQ and EEG pre-post comparisons were analyzed using paired-sample tests selected according to the normality of each variable, with paired t-tests used for normally distributed data and Wilcoxon signed-rank tests used for non-normal data. Given the randomized crossover design, autonomic and SKNA outcomes were analyzed using linear mixed-effects models (LMMs) to account for within-subject correlations. The models included Time (PRE vs. POST), Intervention (RB vs. PRB), and their Interaction as fixed effects, with Subject as a random intercept. To evaluate the robustness of the crossover design, Period and Sequence effects were initially included as fixed effects to check for potential carryover. Post-hoc pairwise comparisons were conducted to examine specific within-group changes from PRE to POST. To control the false discovery rate across multiple post-hoc within-condition comparisons of autonomic and SKNA parameters, the Benjamini–Hochberg procedure was applied. Both unadjusted *p*-values and adjusted *q*-values are reported, and *q* < 0.05 was considered statistically significant for these comparisons. Pearson or Spearman correlation coefficients were used to explore associations between changes (Δ) in EEG and SKNA parameters where appropriate. Correlation analyses were exploratory and were not corrected for multiple comparisons; therefore, these findings should be interpreted cautiously.

## 3 Results

### 3.1 Rivermead post-concussion symptoms questionnaire

Total symptom scores were significantly reduced following both interventions (*p* < 0.01). Somatic symptom scores were significantly lower after PRB (*p* < 0.05), whereas no significant pre-post change was observed after RB (*p* > 0.05). In contrast, emotional symptom scores were significantly lower after RB (*p* < 0.05), whereas no significant pre-post change was observed after PRB (*p* > 0.05). Cognitive symptom scores were significantly lower following both interventions (*p* < 0.05) (Table 1).

**Table 1:** RPQ scores.

Outcome	RB		PRB	
	Pre	Post	Pre	Post
Total score	20.70 ± 12.54 (13.12–28.28)	12.35 ± 4.57 (9.58–15.12)^**^	20.60 ± 11.66 (13.56–27.64)	7.95 ± 3.09 (6.08–9.82)^##^
Somatic	11.10 ± 7.84 (6.36–15.84)	6.20 ± 2.13 (4.91–7.49)	10.75 ± 6.93 (6.56–14.94)	3.95 ± 1.70 (2.92–4.98)^#^
Cognitive	6.75 ± 2.43 (5.28–8.22)	1.55 ± 1.00 (0.95–2.15)^*^	4.50 ± 2.26 (3.13–5.87)	1.10 ± 0.59 (0.74–1.46)^#^
Emotional	4.85 ± 3.23 (2.90–6.80)	3.10 ± 1.41 (2.25–3.95)^*^	5.35 ± 3.50 (3.24–7.46)	3.90 ± 1.48 (3.00–4.80)

^*^p < 0.05 in RB group; ^**^p < 0.01 in RB group; ^#^*p* < 0.05 in PRB group; ^##^*p* < 0.01 in PRB group.

### 3.2 Electroencephalographic (EEG)

**Figure 3:**
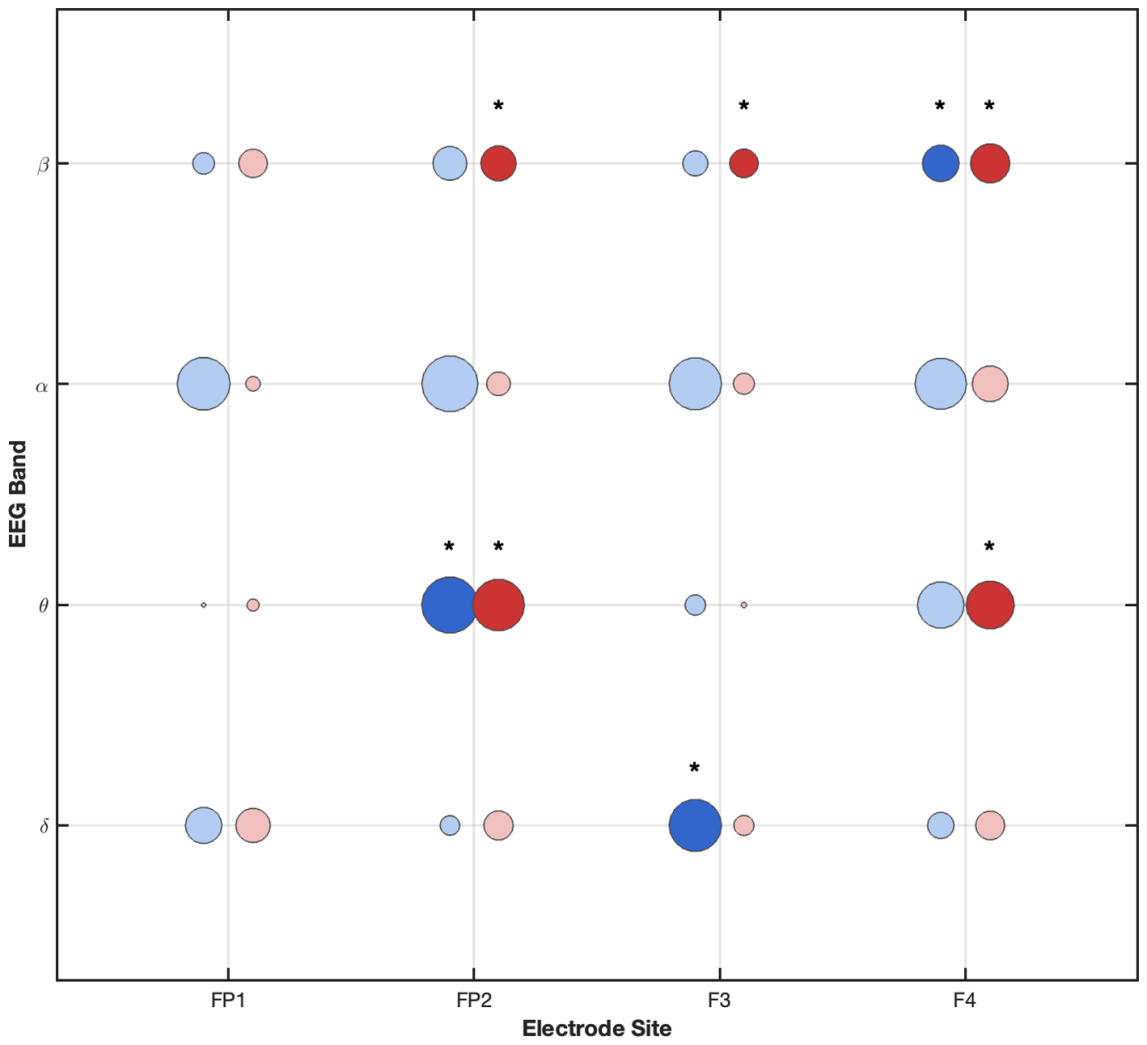
Pre–post EEG changes following RB and PRB.

Pre–post EEG changes were observed in prefrontal and frontal regions under both intervention conditions, with distinct spatial and spectral patterns. Following PRB, significant EEG power changes were observed at multiple sites (*p* < 0.05), specifically in the *β*-band at Fp2, F3, and F4, and in the *θ*-band at Fp2 and F4. In comparison, significant EEG power changes following RB were more limited, primarily involving the *β*-band at F4, *θ*-band at Fp2, and *δ*-band at F3. Notably, at electrode sites where significant pre–post changes were observed under both conditions, the magnitude of change was greater following RB than following PRB (Figure 3).

Bubble size denotes the mean pre-post difference. Color indicates the intervention condition and statistical significance: dark blue, significant pre–post change following RB; light blue, non-significant pre–post change following RB; red, significant pre–post change following PRB; pink, non-significant pre–post change following PRB.

### 3.3 Autonomic function

The effects of intervention type and time on autonomic function were analyzed using linear mixed-effects models (Table 2). Significant main effects of intervention type were observed for aSKNA, burst threshold, and burst amplitude (all *p* < 0.001). Significant time effects were found for aSKNA, burst threshold, burst duration, and burst area (all *p* < 0.05). No significant interaction effects between intervention type and time were detected for any autonomic parameter (all *p* > 0.05).

**Table 2:** Fixed effects from linear mixed-effects models for autonomic parameters.

Outcome	Effect	*F*	*p*-value	Partial *η*^2^
aSKNA	Type	66.52	<0.001^*^	0.509
	Time	12.47	0.001^*^	0.163
	Type × Time	0.77	0.384	0.012
Burst threshold	Type	24.14	<0.001^*^	0.274
	Time	13.96	<0.001^*^	0.179
	Type × Time	0.78	0.382	0.012
Burst frequency	Type	0.16	0.695	0.002
	Time	0.21	0.649	0.003
	Type × Time	1.25	0.269	0.019
Burst duration	Type	0.25	0.616	0.004
	Time	4.47	0.037^*^	0.065
	Type × Time	0.02	0.893	0
Burst amplitude	Type	35.97	<0.001^*^	0.36
	Time	2.41	0.126	0.036
	Type × Time	2.9	0.094	0.043
Burst area	Type	0.96	0.329	0.015
	Time	25.21	<0.001^*^	0.282
	Type × Time	1.32	0.254	0.02

Following FDR correction, significant pre–post changes were observed in heart rate, total power, LF/HF ratio, and burst threshold in the RB condition, as well as in heart rate, total power, burst threshold, and burst frequency in the PRB condition (all *q* < 0.05). No significant changes were observed for aSKNA, burst duration, burst amplitude, or total burst area after correction (all q ≥ 0.05). Within-group analyses are presented for descriptive purposes and should be interpreted in the context of the overall mixed-effects model (Table 3).

**Table 3:** Changes in autonomic parameters following RB and PRB.

Parameter	Group	PRE	POST	Δ	*p*-value	*q*-value (FDR)
HR (bpm)	RB	68.43 ± 15.72	65.13 ± 11.70^*^	−3.3	2.13 × 10^−8^	3.83 × 10^−7^
	PRB	63.35 ± 9.45	59.93 ± 11.07^*^	−3.42	9.10 × 10^−5^	5.46 × 10^−4^
TP	RB	3,004.01 ± 1,784.23	4,194.04 ± 1201.63^*^	1190.03	1.11 × 10^−5^	9.99 × 10^−5^
	PRB	3,848.02 ± 1,957.66	4,065.54 ± 1,168.73^*^	217.52	0.016	0.036
LF/HF	RB	1.54 ± 0.40	1.72 ± 0.28^*^	0.18	0.002	0.006
	PRB	1.51 ± 0.51	1.70 ± 0.55	0.19	0.67	0.709
aSKNA (μV)	RB	0.81 ± 0.25	1.04 ± 0.36	0.23	0.941	0.941
	PRB	1.28 ± 0.36	1.25 ± 0.32	−0.03	0.09	0.135
Burst threshold (μV)	RB	0.94 ± 0.36	1.19 ± 0.56^*^	0.25	0.007	0.018
	PRB	1.30 ± 0.40	1.70 ± 0.52^*^	0.4	0.002	0.006
Burst frequency (times/min)	RB	3.74 ± 2.30	4.01 ± 1.91	0.27	0.086	0.135
	PRB	3.71 ± 1.95	4.39 ± 2.30^*^	0.68	2.57 × 10^−4^	0.00116
Burst duration (%)	RB	18.05 ± 12.58	25.87 ± 15.67	7.82	0.191	0.246
	PRB	19.55 ± 12.25	28.33 ± 23.73	8.78	0.273	0.307
Burst amplitude (μV)	RB	1.05 ± 0.38	1.44 ± 0.61	0.39	0.233	0.28
	PRB	1.96 ± 0.65	1.76 ± 0.62	−0.2	0.051	0.092
Total burst area (μV·s)	RB	0.18 ± 0.11	0.80 ± 0.60	0.62	0.031	0.062
	PRB	0.21 ± 0.17	0.59 ± 0.68	0.38	0.125	0.173

Δ indicates the change from PRE to POST. Asterisks indicate statistical significance after false discovery rate (FDR) correction using the Benjamini–Hochberg procedure (*q* < 0.05). The Benjamini–Hochberg adjustment was applied only to the post-hoc within-condition comparisons reported in this table. The asterisk (*) indicates significance after FDR correction (*p* < 0.05).

### 3.4 Correlation between sympathetic nerve activity and EEG

Pearson correlation analysis revealed correlations between changes in alpha wave relative power in the prefrontal region and changes in SKNA burst patterns after PRB: enhanced activity in the left prefrontal cortex (Fp1) showed a moderate negative correlation with suppressed SKNA bursts (decreased frequency and shorter duration) (*p* < 0.05). In contrast, enhanced activity in the right prefrontal cortex (Fp2) and bilateral DLPFC (F3, F4) positively correlated with significant increases in burst threshold, frequency, and amplitude (*p* < 0.05), accompanied by decreases in burst duration and total area (*p* < 0.05) (Table 4).

**Table 4:** Correlations of EEG and SKNA changes post-PRB.

EEG electrode site	Δaskna	ΔBurst threshold (μV)	ΔBurst frequency (times/min)	ΔBurst duration (%)	ΔBurst amplitude (μV)	ΔTotal burst area (μV·S)
ΔFp1	0.236	0.378	−0.499^*^	−0.686^**^	0.391	−0.489^*^
ΔFp2	0.659^**^	0.675^**^	−0.037	−0.287	0.690^**^	−0.482^*^
ΔF3	0.474^*^	0.592^**^	−0.239	−0.553^*^	0.604^**^	−0.587^*^
ΔF4	0.643^**^	0.648^**^	−0.003	−0.292	0.658^**^	−0.432

Δ = POST–PRE; ^*^p < 0.05; ^**^p < 0.01.

## 4 Discussion

The present study examined the effects of resonance breathing (RB) and pelvic floor resonance breathing (PRB) on clinical symptoms, autonomic function, and electrocortical activity in athletes with sports-related concussion (SRC). Overall, both interventions were associated with improvements in symptom severity, modulation of heart rate variability (HRV), and changes in EEG patterns, although distinct response profiles were observed between RB and PRB.

### 4.1 Symptom improvement

The results of this study suggest that slow breathing interventions were associated with reductions in persistent SRC symptoms, with RB and PRB showing somewhat distinct patterns across symptom dimensions. The comparable effects of both interventions on total symptom scores indicate that resonance breathing may represent a useful non-pharmacological approach for managing SRC symptoms.

RB and PRB showed different patterns of improvement across symptom domains. PRB appeared to have advantages in reducing somatic symptoms such as headache and dizziness. This effect may be related to its influence on basal sympathetic tone and the organization of sympathetic nerve burst patterns. Reduced sympathetic tension may help alleviate abnormal peripheral vasoconstriction and improve cerebral blood flow, which may in turn be associated with relief of physical discomfort. In addition, the coordinated pelvic floor–diaphragmatic activation in PRB may contribute to improvements in cervicogenic or somatically related symptoms by influencing thoracoabdominal pressure dynamics and spinal stability.

In contrast, RB appeared to show stronger associations with improvements in emotional symptoms. The enhancement of prefrontal theta activity may provide a potential neural basis for this observation. The prefrontal cortex, particularly the dorsolateral prefrontal cortex, plays a key role in emotional regulation. Increased theta oscillations following RB may reflect enhanced top-down regulatory processes involving this region and its interactions with limbic structures such as the amygdala. This may be related to improvements in emotional symptoms including anxiety and irritability. The focused and steady breathing pattern inherent to RB may also provide a supportive behavioral context for emotional regulation.

Notably, cognitive symptoms improved after both interventions. This finding is consistent with our EEG results. We observed increased prefrontal theta activity under both conditions. Prefrontal theta is associated with attention and cognitive control (Cavanagh and Frank, 2014). Although RB and PRB use different approaches, both may promote neural states that support cognitive recovery. This shared effect suggests that the underlying mechanism may be related to their ability to modulate autonomic balance and prefrontal function.

### 4.2 Autonomic modulation

The results of this study suggest that both RB and PRB were associated with changes in autonomic nervous function in athletes with SRC, although the patterns of response differed between the two interventions. Under both conditions, HR decreased and total HRV power increased. This pattern is consistent with previous findings indicating that slow breathing may enhance cardiopulmonary coupling and vagally mediated regulation.

Following RB, increases in both the LF/HF ratio and total SKNA burst area were observed. These findings may reflect changes in autonomic dynamics rather than a simple reduction in sympathetic activity. One possible explanation is that resonance breathing at approximately 6 breaths per minute may improve the coupling between respiratory rhythms and cardiovascular oscillations (Tatschl and Schwerdtfeger, 2022), which could enhance the coordination of autonomic signaling. However, given the ongoing debate regarding the physiological interpretation of LF, HF, and particularly the LF/HF ratio, these results should be interpreted with caution.

In contrast, PRB was associated with increases in SKNA burst threshold and burst frequency, along with a reduction in heart rate. This pattern may suggest a reorganization of sympathetic activity rather than generalized suppression. An increased burst threshold may indicate reduced tonic firing, while the concurrent increase in burst frequency may reflect preserved or enhanced phasic responses (Hagbarth et al., 1972). This combination may be consistent with a shift from a more diffuse sympathetic pattern toward a more structured and pulsatile mode of activity (Kusayama et al., 2019).

The mechanisms underlying this pattern remain unclear. One possible explanation is that the coordinated activation of the pelvic floor muscles, diaphragm, and deep trunk musculature during PRB may increase cyclical fluctuations in intra-abdominal and thoracoabdominal pressure. These changes could influence venous return and baroreceptor-mediated signaling, which may in turn affect autonomic regulation (Schelegle, 2003). In addition, pelvic floor activation may increase sensorimotor input and coordination demands, potentially enhancing interactions between somatic and autonomic systems (Talasz et al., 2010; Talasz et al., 2022). However, these interpretations remain speculative and require further investigation.

Overall, these findings suggest that both RB and PRB may support physiological recovery following SRC, although potentially through different pathways. RB may be more related to enhancing rhythmic coordination within the autonomic nervous system, whereas PRB may be associated with more specific modulation of sympathetic discharge patterns.

### 4.3 EEG changes

Different EEG frequency bands are associated with distinct neural functions. Alpha activity is commonly linked to relaxation and reduced mental effort and is often observed during calm states (Cahn and Polich, 2006; Klimesch, 1999). It may also be associated with increased parasympathetic activity, although this relationship can vary across tasks and physiological states (Doufesh et al., 2014; Kawashima et al., 2024). Beta activity is related to active thinking, attention, and cortical arousal, and higher beta power is often observed during states of alertness or mental stress (Engel and Fries, 2010; Knyazev, 2007). Theta activity is associated with cognitive processing and internal attention and has also been linked to states of deep relaxation and emotional regulation (Cavanagh and Frank, 2014; Klimesch, 1999).

The EEG findings of this study suggest that respiratory interventions may influence central regulatory processes in SRC. Both RB and PRB were associated with changes in activity in the prefrontal cortex and dorsolateral prefrontal cortex, although their spatial distributions and spectral characteristics differed. Overall, changes in theta, beta, and delta band power may indicate that these interventions influence attentional control, cognitive engagement, and cortical state regulation in different ways.

One of the most consistent findings was the increase in prefrontal theta activity. Theta oscillations in this region are commonly associated with focused attention, cognitive control, and self-regulation. During both RB and PRB, participants were required to maintain sustained attention to breathing rhythms and bodily states, which may impose a stable cognitive demand. From this perspective, the observed increase in theta activity may reflect enhanced attentional regulation following the interventions. This interpretation is consistent with the improvement in cognitive symptoms observed in both conditions.

More extensive EEG changes were observed following PRB. This may be related to the greater complexity of the breathing pattern. PRB involves coordinated activation of the pelvic floor muscles in addition to diaphragmatic breathing. The combined activation of the pelvic floor, diaphragm, and trunk musculature may increase fluctuations in thoracoabdominal pressure, which could influence venous return and cerebral blood flow. These physiological changes may also interact with autonomic regulation through baroreceptor-mediated mechanisms (Conde et al., 2022). In addition, the requirement to coordinate breathing with pelvic floor muscle activation may increase cognitive and sensorimotor demands. Together, these factors may contribute to the more pronounced changes in theta and beta activity observed in the prefrontal cortex during PRB. However, these interpretations remain speculative.

In contrast, RB showed a different pattern of EEG changes. The task is relatively simple and repetitive, which may support a more stable attentional and emotional state. This may be consistent with its stronger association with improvements in emotional symptoms (Liang et al., 2022). The differences observed between RB and PRB do not necessarily indicate that one intervention is superior. Instead, they may reflect different modes of neuromodulation. RB may be more related to the stabilization of attentional and emotional states (Risqiwati et al., 2024), whereas PRB may involve broader sensorimotor and autonomic engagement.

In summary, the findings suggest that breathing-related EEG changes may reflect modulation of arousal, attention, and cognitive control processes. Although the current study does not allow for definitive conclusions regarding underlying mechanisms, the results support the possibility that different breathing patterns are associated with distinct electrophysiological responses.

### 4.4 Brain and autonomic nervous system correlations

Pearson correlation analysis indicated that, following the PRB intervention, changes in prefrontal EEG activity were associated with changes in SKNA. Further analysis showed that an increase in alpha power at the Fp1 electrode site was moderately negatively correlated with SKNA burst activity. Previous studies have reported that enhanced alpha oscillations are typically associated with improved cortical inhibitory control (Knyazev, 2007). In addition, the Fp1 region is known to play an important role in intrinsic attention and emotion regulation (Viviani, 2013). Based on these findings, the observed correlations may suggest that PRB is associated with enhanced inhibitory regulatory processes in this region, and potentially with reduced sympathetic overactivation.

Consistent with these findings, activity in the right frontal pole (Fp2) and the bilateral dorsolateral prefrontal cortex (DLPFC) showed positive correlations with changes in SKNA burst characteristics. Increased alpha power in these regions was associated with higher burst thresholds, frequencies, and amplitudes, along with reduced burst duration and total area. This pattern may indicate a shift in the organization of sympathetic activity. It is possible that burst characteristics with shorter durations and higher amplitudes reflect a more efficient mode of sympathetic regulation. As key regions involved in higher-order cognitive control, the right prefrontal cortex and DLPFC, when showing increased activity, may be associated with improved regulation of sympathetic responses (Friedman and Robbins, 2022). This may be reflected as more controlled initiation and termination of sympathetic bursts following PRB training.

These findings also suggest potential functional differentiation within the prefrontal regulatory network. The left prefrontal region may be more involved in suppressing background sympathetic activity, whereas the right prefrontal cortex and DLPFC may play a greater role in shaping burst patterns. Taken together, this may reflect a transition from a more continuous and diffuse sympathetic state toward a more transient and regulated pattern. However, these interpretations remain preliminary and require further empirical validation.

### 4.5 Limitations

This study has the following limitations: First, the sample size was relatively small. Although the crossover design improved the sensitivity of within-group comparisons to some extent, the relevant conclusions still need to be validated in larger samples. Second, the sample consisted only of male rugby athletes, which limits the generalizability of the findings to female athletes, other sports, and non-athletic populations. Third, this study included multiple electroencephalography and autonomic nervous system indicators, which may increase the risk of Type I errors; therefore, the results should be interpreted with caution. Fourth, the correlation analyses were exploratory and were not corrected for multiple comparisons. Finally, the lack of a sham or non-intervention control condition limits the ability to make causal inferences. The observed changes may be influenced by time factors, expectancy effects, repeated testing, or nonspecific recovery processes. There were differences in the time from injury to participant enrollment. Future studies should be conducted with larger samples and under stricter experimental control conditions to further validate the findings of this study.

## 5 Conclusion

This trial suggests that both RB and PRB were associated with reductions in symptoms in athletes with SRC. They were also associated with changes in neurophysiological function, although the patterns differed between interventions. RB appeared more related to emotional and autonomic regulation, whereas PRB appeared more related to somatic symptoms and sympathetic activity. These findings suggest that respiratory exercises may represent a potentially useful adjunctive approach for concussion rehabilitation.

## Data Availability

The original contributions presented in the study are included in the article/supplementary material, further inquiries can be directed to the corresponding author.
